# Macrophage Notch1 inhibits TAK1 function and RIPK3-mediated hepatocyte necroptosis through activation of β-catenin signaling in liver ischemia and reperfusion injury

**DOI:** 10.1186/s12964-022-00901-8

**Published:** 2022-09-16

**Authors:** Dongwei Xu, Xiaoye Qu, Yizhu Tian, Zhao Jie, Zhifeng Xi, Feng Xue, Xueyun Ma, Jianjun Zhu, Qiang Xia

**Affiliations:** 1 Department of Liver Surgery, Renji Hospital, Shanghai Jiaotong University School of Medicine, Shanghai, 200127 China; 2 Department of General Surgery, Pukou Branch of Jiangsu Province Hospital, Nanjing Medical University, Nanjing, 211166 China; 3 Shanghai Key Laboratory of Regulatory Biology, Institute of Biomedical Sciences and School of Life Sciences, East China Normal University, Shanghai, 200241 China

**Keywords:** Macrophage, Innate immunity, Notch1, Necroptosis, TAK1

## Abstract

**Background:**

Notch signaling is highly conserved and critically involved in cell differentiation, immunity, and survival. Activation of the Notch pathway modulates immune cell functions during the inflammatory response. However, it remains unknown whether and how the macrophage Notch1 may control the innate immune signaling TAK1, and RIPK3-mediated hepatocyte necroptosis in liver ischemia and reperfusion injury (IRI). This study investigated the molecular mechanisms of macrophage Notch1 in modulating TAK1-mediated innate immune responses and RIPK3 functions in liver IRI.

**Methods:**

Myeloid-specific Notch1 knockout (Notch1^M−KO^) and floxed Notch1 (Notch1^FL/FL^) mice (n = 6/group) were subjected to 90 min partial liver warm ischemia followed by 6 h of reperfusion. In a parallel in vitro study, bone marrow-derived macrophages (BMMs) were isolated from these conditional knockout mice and transfected with CRISPR/Cas9-mediated β-catenin knockout (KO) vector followed by LPS (100 ng/ml) stimulation.

**Results:**

IR stress-induced Notch1 activation evidenced by increased nuclear Notch intracellular domain (NICD) expression in liver macrophages. Myeloid Notch1 deficiency exacerbated IR-induced liver damage, with increased serum ALT levels, macrophage/neutrophil accumulation, and proinflammatory cytokines/chemokines production compared to the Notch1^FL/FL^ controls. Unlike in the Notch1^FL/FL^ controls, Notch1^M−KO^ enhanced TRAF6, TAK1, NF-κB, RIPK3, and MLKL but reduced β-catenin activation in ischemic livers. However, adoptive transfer of lentivirus β-catenin-modified macrophages markedly improved liver function with reduced TRAF6, p-TAK1, RIPK3 and p-MLKL in IR-challenged livers. Moreover, disruption of RIPK3 in Notch1^M−KO^ mice with an in vivo mannose-mediated RIPK3 siRNA delivery system diminished IR-triggered hepatocyte death. In vitro studies showed that macrophage NICD and β-catenin co-localized in the nucleus, whereby β-catenin interacted with NICD in response to LPS stimulation. Disruption of β-catenin with a CRISPR/Cas9-mediated β-catenin KO in Notch1^FL/FL^ macrophage augmented TRAF6 activation leading to enhanced TAK1 function. While CRISPR/Cas9-mediated TRAF6 KO in Notch1^M−KO^ macrophage inhibited RIPK3-mediated hepatocyte necroptosis after co-culture with primary hepatocytes.

**Conclusions:**

Macrophage Notch1 controls TAK1-mediated innate immune responses and RIPK3-mediated hepatocyte necroptosis through activation of β-catenin. β-catenin is required for the macrophage Notch1-mediated immune regulation in liver IRI. Our findings demonstrate that the macrophage Notch1-β-catenin axis is a crucial regulatory mechanism in IR-triggered liver inflammation and provide novel therapeutic potential in organ IRI and transplant recipients.

**Video abstract**

**Supplementary Information:**

The online version contains supplementary material available at 10.1186/s12964-022-00901-8.

## Background

Liver ischemia and reperfusion injury (IRI) is one of the staple causes of hepatic dysfunction or failure in liver transplants [1, 2]. Liver inflammation induced by IRI involves oxidative stress and endoplasmic reticulum (ER) stress-mediated inflammatory responses [3, 4]. Liver macrophages are the key components of the hepatic innate immune system. Liver IRI activates liver macrophage cells and promotes the generation of reactive oxygen species (ROS) and cytokines, such as IL-6 and TNF-α. By recognizing exogenous danger signals containing pathogen-derived molecular patterns (PAMPs) or endogenous molecules damage-associated molecular patterns (DAMPs) that are released from ruptured cells in inflammation, sterile inflammatory responses are activated [4, 5].

Four Notch receptors and five Notch ligands containing Jagged1, Jagged2, DLL1, DLL3, and DLL4 have been reported [6]. The Notch intracellular domain (NICD) is released when Jagged1 proteins bind to the Notch extracellular domain inducing proteolytic cleavage. Jagged1 (JAG1) plays a crucial role in the cell development process. Notch1 and JAG1 ligand Overexpression can improve cell survival in liver regeneration [7]. Furthermore, Notch signaling mediates the homeostasis of innate immune [8, 9]. Inhibition of the RBP-J promotes cell death and inflammatory injury, inducing increased inflammatory injury [10]. Although Notch signaling is proved to relate with hepatocellular protection in liver inflammation, further underlying mechanism containing Notch signaling and liver inflammatory responses need to be elucidated.

Transforming growth factor β-activated kinase1 (TAK1) plays a critical role in the process of cellular inflammatory pathways [11]. For instance, in TNF-α and Toll-like receptor (TLR) ligand pathways, TAK1 is recruited by K63 ubiquitination chain-modified signaling complexes to promote its activation [12]. TAK1 is activated to phosphorylate the downstream components such as JNK, p38, and ERK to stimulate NF-κB and MAPK pathways [13]. TAK1 deficiency in hepatocytes and cholangiocytes leads to liver cell death, inflammation, fibrosis, and HCC [14]. In addition, TAK1 liver deficiency in mice presents similarity to the gene-expression signature of human HCC. However, the crosstalk between Notch1 and TAK1 remains unknown in liver IRI.

Receptor-interacting serine/threonine-protein kinase 3(RIPK3) and its downstream molecule mixed-lineage kinase domain like protein (MLKL) have been strongly proved to mediate necroptosis, an essential form of programmed cell death [15]. On activation, RIPK3 phosphorylates MLKL, which plays a critical role in necroptosis. Necroptosis is highly immunogenic and may contribute to inflammation and carcinogenesis when sustained. RIPK3-dependent hepatocyte necroptosis is activated in IRI liver, but the particular role of RIPK3-dependent signaling in innate immune response remains elusive. In this study, we for the first time investigated the roles and molecular mechanisms of macrophage Notch1 in modulating TAK1-mediated innate immune responses and RIPK3 functions in liver IRI.

## Methods

### Animals

The Notch1^M−KO^ mice were generated as described [9]. A standard protocol was used to perform mouse genotyping with primers shown in the JAX Genotyping protocols database. All animal studies were approved by the Institutional Animal Care and Use Committees of Renji Hospital and Shanghai Jiaotong University.

### Mouse liver IRI model

An established model of mice 70% warm hepatic ischemia (90 min) followed by reperfusion (6 h) was used [9, 16]. Some mice have been injected with the bone marrow-derived macrophages (BMMs, 5 × 10^6^ cells in PBS/mouse) transfected with lentivirus-expressing β-catenin (Lv-β-catenin) or control lentivirus (GFP) via the tail vein 24 h before ischemia. Some mice were injected with RIPK3 siRNAs or non-specific (NS) control siRNA (2 mg/kg) (Santa Cruz Biotechnology, Santa Cruz, CA) mixed with mannose-conjugated polymers (Polyplus transfection™, Illkirch, France) according to the manufacturer’s instructions 4 h before ischemia.

### Hepatocellular function assay

The serum alanine aminotransferase (sALT) and aspartate aminotransferase (AST) were measured by ALT and AST kit (ThermoFisher, Waltham, MA) according to the manufacturer’s instructions.

### Histology, immunohistochemistry, and immunofluorescence staining

The paraffin-embedded liver sections (5-μm) were stained with hematoxylin and eosin (H&E). The Suzuki’s criteria were used to grade the severity of IRI [17]. Liver macrophages were indicated by primary CD11b + rat monoclonal antibodies (mAb) using immunofluorescence staining, and the neutrophils were measured using primary Ly6G rat mAb (Invitrogen, San Diego, CA) by immunohistochemistry (IHC) staining. The RIPK3 in the mice liver was detected by IHC using RIPK3 mAb (Abcam, Cambridge, MA). The primary mouse NICD (Santa Cruz Biotechnology) mAb and rabbit β-catenin (Cell Signaling Technology) mAb were used for the double immunofluorescence staining according to the manufacturer’s instructions. Images for immunofluorescence staining were captured using a fluorescence microscope (Keyence BZ-X810, Osaka, Japan) and analyzed using Image-pro Plus software. Positive cells were counted blindly in 10 HPF/section (× 200).

### Real-time reverse transcription-PCR

The RT-PCR was performed as described [18]. Total RNA was purified from liver tissue or cell cultures using RNeasy Mini Kit (Qiagen, Chatsworth, CA) according to the manufacturer’s instructions. Quantitative real-time PCR was carried out using the QuantStudio 3 (Applied Biosystems by ThermoFisher Scientific). The following were added in the final reaction volume of 25 μl: 1 × SuperMix (Platinum SYBR Green qPCR Kit; Invitrogen) cDNA and 10 μM of each primer. Amplification conditions were: 50 °C (2 min), 95 °C (5 min), followed by 40 cycles of 95 °C (15 s) and 60 °C (30 s). Primer sequences used to amplify TNF-α, IL-1β, CCL-2, and CXCL-10 were shown in Additional file 2: Table S1.

### Western blot analysis

Protein was extracted from liver tissue or cell cultures as described [19]. The NICD, Bax, Bcl-2, c-caspase-3, TRAF6, p-TAK1 (Ser412), TAK1, p-P65 (Ser536), P65, RIPK3, p-MLKL (Ser345), Lamin B2, β-actin (Cell Signaling Technology) were used. The Western ECL substrate mixture (Bio-Rad) was used to image with the iBright FL1000 (ThermoFisher Scientific). See Additional file 1: Materials.

### Statistical analysis

Data are expressed as mean ± SD and analyzed by Permutation t-test and Pearson correlation. Per comparison, two-sided *p* values less than 0.05 were considered statistically significant. Multiple group comparisons were made using one-way ANOVA followed by Bonferroni’s post hoc test. When groups showed unequal variances, we applied Welch’s ANOVA to make multiple group comparisons. All analyses were used by SAS/STAT software, version 9.4.

Additional materials and methods are included in supplemental materials and methods.

## Results

### Disruption of myeloid-specific Notch1 aggravates IR-induced liver injury and increases macrophage accumulation and proinflammatory mediators in IR-stressed liver

**Fig. 1:**
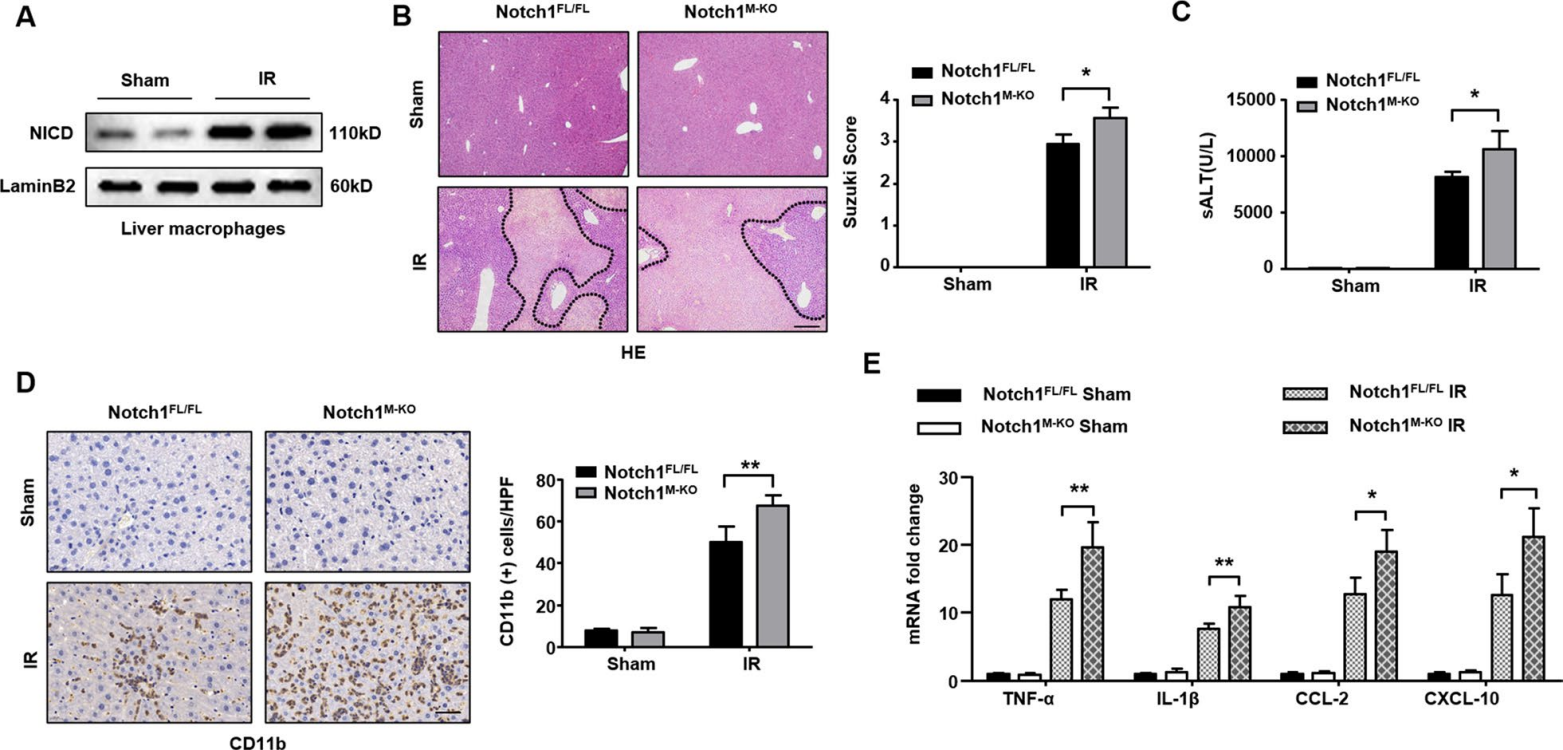
Disruption of myeloid-specific Notch1 aggravates IR-induced liver injury and increases macrophage accumulation and proinflammatory mediators in IR-stressed liver. The Notch1^FL/FL^, Notch1^M−KO^ and WT mice were subjected to 90 min of partial liver warm ischemia, followed by 6 h of reperfusion. **A** The nuclear NICD expression was detected in liver macrophage cells from IR-stressed livers by Western blot assay. Representative of three experiments. **B** Representative histological staining (H&E) of ischemic liver tissue (n = 4–6 mice/group) and Suzuki’s histological score. Scale bars, 200 μm. **C** Liver function in serum samples was evaluated by serum ALT levels (IU/L) (n = 4–6 samples/group). **D** Immunohistochemistry staining of CD11b^+^ macrophages in ischemic livers (n = 4–6 mice/group). Quantification of CD11b^+^ macrophages, Scale bars, 40 μm. **E** Quantitative RT-PCR analysis of TNF-α, IL-1β, CCL-2 and CXCL-10 mRNA levels in ischemic livers (n = 3–4 samples/group). All data represent the mean ± SD. *p < 0.05. **p < 0.01

The myeloid-specific Notch1-deficient (Notch1^M−KO^), Notch1-proficient (Notch1^FL/FL^), and WT mice were subjected to 90 min of warm ischemia followed by 6 h of reperfusion. The liver macrophages were isolated from WT groups. First, we found that IR stress-induced increased nuclear Notch intracellular domain (NICD) in liver macrophages (Fig. 1A). The Notch1^M−KO^ livers showed severe edema, sinusoidal congestion, and necrosis than the Notch1^FL/FL^ livers, which showed mild to moderate edema, sinusoidal congestion, and narrow hepatocellular necrosis (Fig. 1B). The liver damage was evaluated by Suzuki’s histological grading of liver IRI. The hepatocellular functions are measured by the serum ALT (sALT) levels (IU/L). Myeloid Notch1 deficiency significantly augmented sALT levels at 6 h post-liver reperfusion in the Notch1^M−KO^ mice compared to the Notch1^FL/FL^ controls (Fig. 1C). Moreover, the Notch1^M−KO^ ischemic livers showed increased accumulation of CD11b^+^ macrophages (Fig. 1D), accompanied by elevated mRNA levels coding for TNF-α, IL-1β, CCL-2, and CXCL-10 in ischemic livers (Fig. 1E), compared to the Notch1^FL/FL^ controls. These results suggest that the Notch1 pathway plays a critical role in IR stress-induced liver inflammation and injury.

### Disruption of myeloid-specific Notch1 exacerbates IR-triggered hepatocyte death

**Fig. 2:**
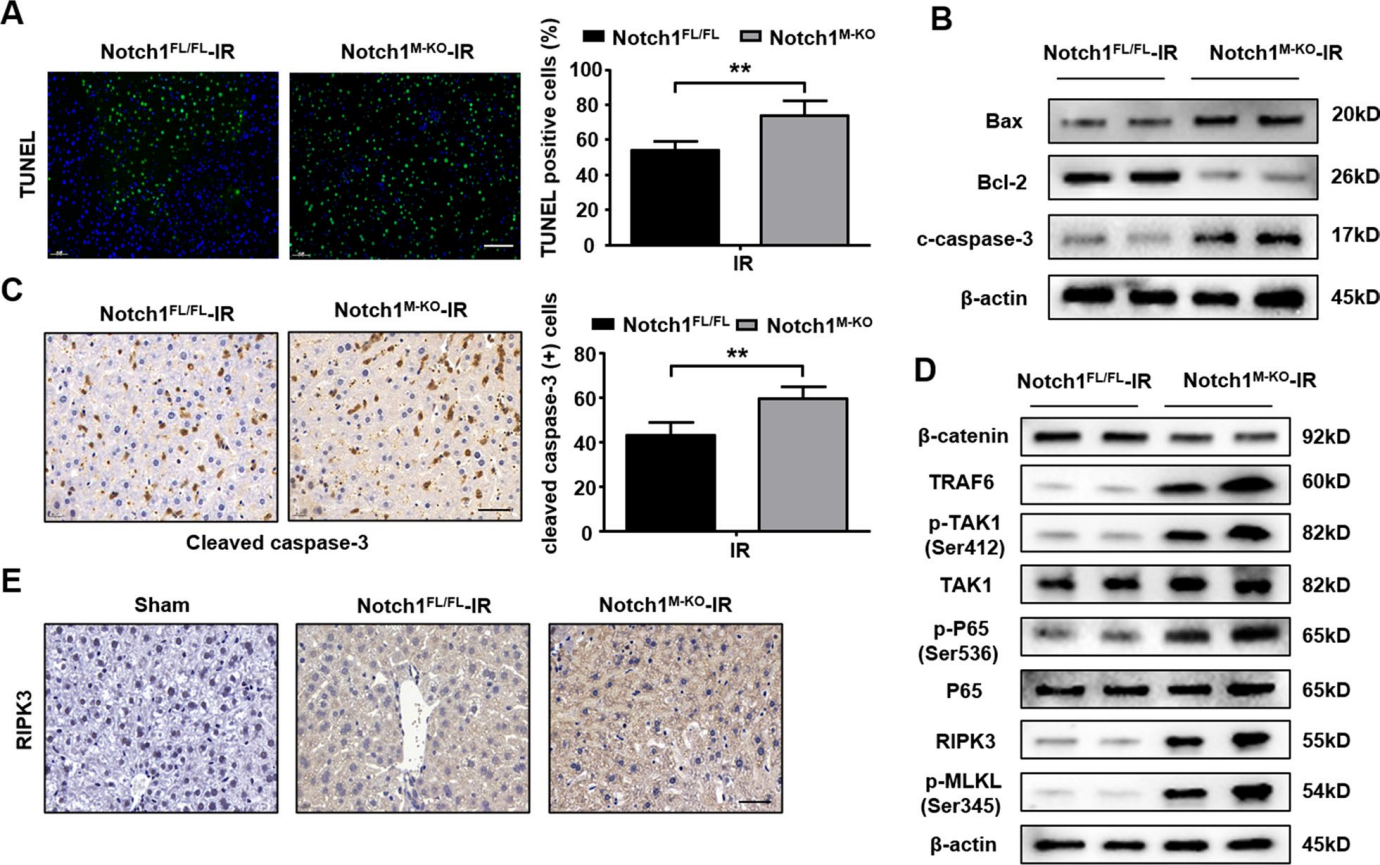
Disruption of myeloid-specific Notch1 exacerbates IR-triggered hepatocyte death. The Notch1^FL/FL^ and Notch1^M−KO^ mice were subjected to 90 min of partial liver warm ischemia, followed by 6 h of reperfusion. **A** TUNEL staining in ischemic livers (n = 4–6 mice/group). Scale bars, 100 μm. **B** Western blot analysis of Bax, Bcl-2, and cleaved caspase-3. Representative of three experiments. **C** Immunohistochemistry staining of cleaved caspase-3 in ischemic livers (n = 4–6 mice/group). Quantification of c-caspase3 positive cells, Scale bars, 40 μm. **D** Western blot analysis of TRAF6, p-TAK1, p-P65, RIPK3 and p-MLKL in Notch1^FL/FL^ and Notch1^M−KO^ mice. Representative of three experiments. **E** Immunohistochemistry staining of RIPK3 in ischemic livers (n = 4–6 mice/group). Scale bars, 40 μm. All data represent the mean ± SD. **p < 0.01

The myeloid-specific Notch1-deficient (Notch1^M−KO^) and Notch1-proficient (Notch1^FL/FL^) mice were subjected to 90 min of warm ischemia followed by 6 h of reperfusion. Immunofluorescence staining revealed increased TUNEL^+^ hepatocytes in Notch1^M−KO^ mice compared to Notch1^FL/FL^ group after IR injury (Fig. 2A). The expression of Bax and cleaved-caspase3 were remarkably increased in Notch1^M−KO^ mice, accompanied by the down-regulated level of Bcl-2 in ischemic livers (Fig. 2B). Similarly, immunohistochemistry staining of cleaved caspase-3 showed increased positive cells counts in Notch1^M−KO^ mice (Fig. 2C). In addition, knockout of myeloid Notch1 in mice down-regulated level of β-catenin, while up-regulated the TRAF6, p-TAK1, p-P65, RIPK3, and p-MLKL expression (Fig. 2D), along with the enhanced level of RIPK3 using immunohistochemistry staining after IR injury (Fig. 2E). These results indicate that disruption of myeloid-specific Notch1 exacerbates IR-triggered hepatocyte death.

### β-catenin is essential for the macrophage Notch1-mediated inflammatory response in liver IRI

**Fig. 3:**
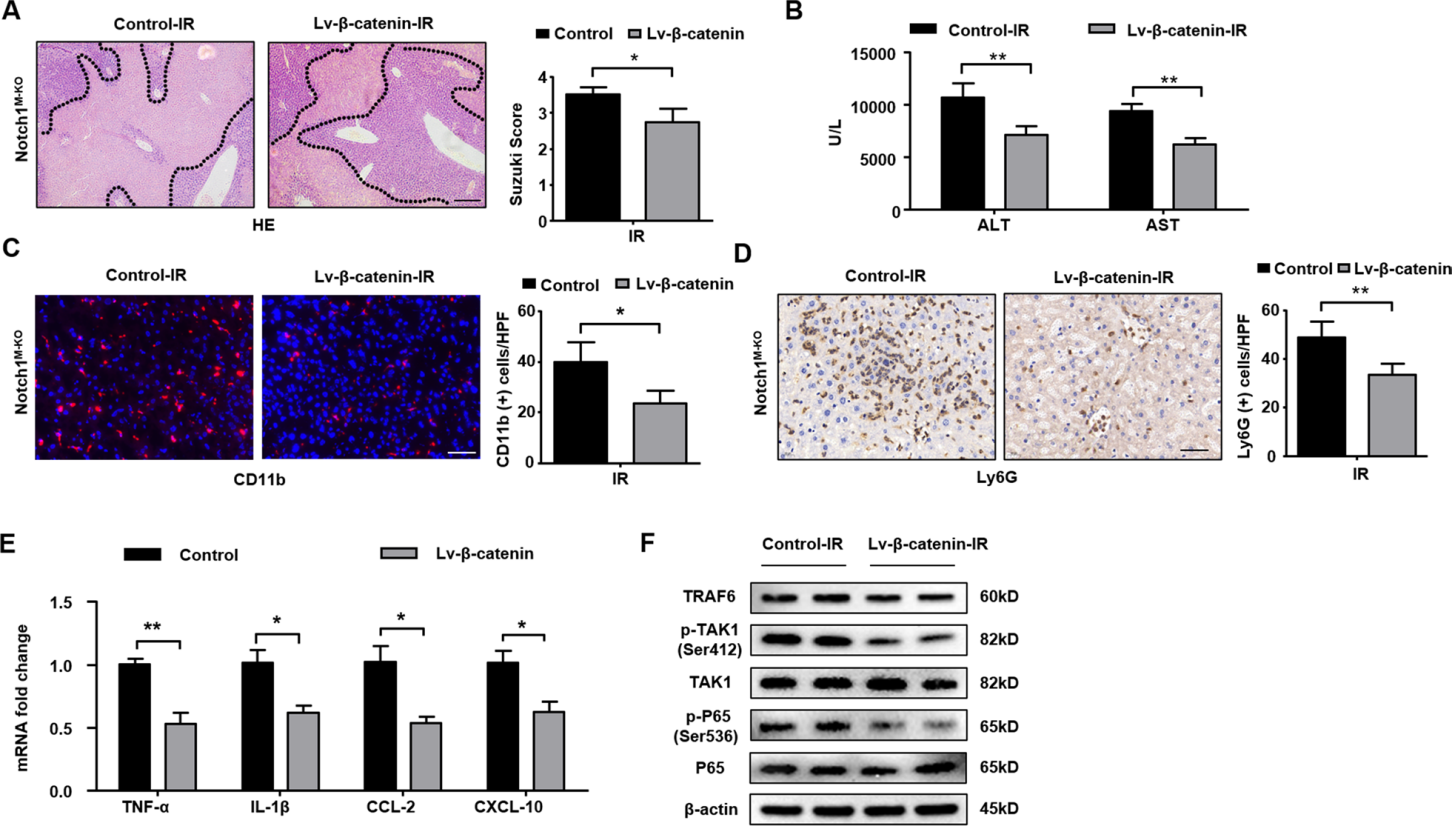
β-catenin is essential for the macrophage Notch1-mediated inflammatory response in liver IRI. The Notch1^M−KO^ mice were injected via tail vein with bone marrow-derived macrophages (BMMs, 5 × 10^6^ cells/mouse) transfected with lentivirus expressing β-catenin (Lv-β-catenin) or GFP control (Lv-GFP) 24 h prior to ischemia. **A** Representative histological staining (H&E) of ischemic liver tissue (n = 4–6 mice/group) and Suzuki’s histological score. Scale bars, 200 μm. **B** Liver function in serum samples was evaluated by serum ALT and AST levels (IU/L) (n = 4–6 samples/group). **C** Immunofluorescence staining of CD11b^+^ macrophages in ischemic livers (n = 4–6 mice/group). Quantification of CD11b^+^ macrophages, Scale bars, 40 μm. **D** Immunohistochemistry staining of Ly6G^+^ neutrophils in ischemic livers (n = 4–6 mice/group). Quantification of Ly6G^+^ neutrophils, Scale bars, 40 μm. **E** Quantitative RT-PCR analysis of TNF-α, IL-1β, CCL-2 and CXCL-10 mRNA levels in ischemic livers (n = 3–4 samples/group). **F** Western blot analysis and relative density ratio of TRAF6, p-TAK1, and p-P65. Representative of three experiments. All data represent the mean ± SD. *p < 0.05. **p < 0.01

To investigate the role of β-catenin in macrophage Notch1-mediated immune regulation in IR-stressed liver, an adoptive transfer of lentivirus β-catenin-modified or control lentivirus-modified macrophages was constructed. As expected, lentivirus β-catenin treatment increased β-catenin expression and reduced TRAF6, p-TAK1, and p-P65 expression in isolated BMMs (Additional file 3: Fig. S1A). The mRNA levels of TNF-α, IL-1β, CCL-2, and CXCL-10 were inhibited after the overexpression of β-catenin in BMM cultures (Additional file 3: Fig. S1B). Interestingly, overexpression of β-catenin in the Notch1^M−KO^ mice alleviated IR-induced liver damage evidenced by decreased Suzuki’s histological score (Fig. 3A) and sALT and sAST levels (Fig. 3B), compared to the control group. Moreover, lentivirus β-catenin treatment in the Notch1^M−KO^ ischemic livers reduced CD11b^+^ macrophage (Fig. 3C) and Ly6G^+^ neutrophil (Fig. 3D) accumulation. The mRNA levels of TNF-α, IL-1β, CCL-2, and CXCL-10 in Notch1^M−KO^ ischemic livers were also inhibited after the overexpression of β-catenin in macrophage (Fig. 3E). Unlike the control group, lentivirus β-catenin treatment reduced TRAF6, p-TAK1, and p-P65 expression (Fig. 3F) in the Notch1^M−KO^ livers. These results demonstrate that β-catenin is a crucial regulator of Notch1-mediated inflammatory response in IR-stress livers.

### Myeloid Notch1 signaling controls immune regulation and hepatocyte death in a RIPK3-dependent manner in IR-stressed liver

**Fig. 4:**
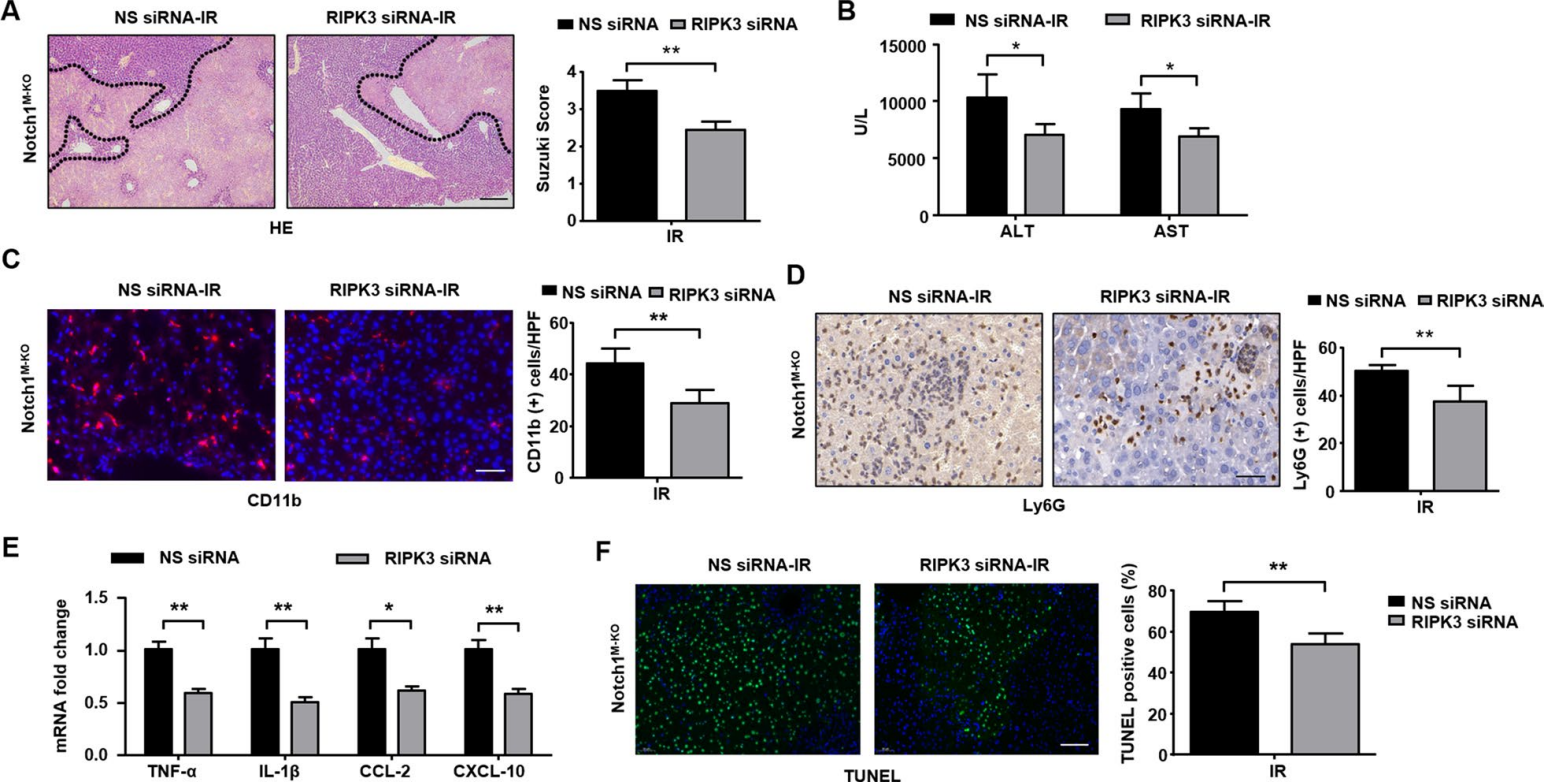
Myeloid Notch1 signaling controls immune regulation and hepatocyte necroptosis in a RIPK3-dependent manner in IR-stressed liver. The Notch1^M−KO^ mice were injected via tail vein with RIPK3 siRNA (2 mg/kg) or non-specific (NS) control siRNA mixed with mannose-conjugated polymers at 4 h prior to ischemia. **A** Representative histological staining (H&E) of ischemic liver tissue (n = 4–6 mice/group) and Suzuki’s histological score. Scale bars, 200 μm. **B** Liver function in serum samples was evaluated by serum ALT and AST levels (IU/L) (n = 4–6 samples/group). **C** Immunofluorescence staining of CD11b^+^ macrophages in ischemic livers (n = 4–6 mice/group). Quantification of CD11b^+^ macrophages, Scale bars, 40 μm. **D** Immunohistochemistry staining of Ly6G^+^ neutrophils in ischemic livers (n = 4–6 mice/group). Quantification of Ly6G^+^ neutrophils, Scale bars, 100 μm. **E** Quantitative RT-PCR analysis of TNF-α, IL-1β, CCL-2 and CXCL-10 mRNA levels in ischemic livers (n = 3–4 samples/group). **F** TUNEL staining in ischemic livers (n = 4–6 mice/group). Results were scored semi-quantitatively by averaging the number of necroptotic cells. All data represent the mean ± SD. *p < 0.05. **p < 0.01

Having found that the RIPK3 functions were augmented in Notch1^M−KO^ mice in liver IRI, we then examined whether RIPK3 is essential in liver inflammation and cellular injury in Notch1-mediated IR-stressed livers. We disrupted RIPK3 in Notch1^M−KO^ livers with an in vivo mannose-mediated RIPK3 siRNA delivery system that delivers explicitly to macrophages by expressing a mannose-specific membrane receptor as previously described [20]. The efficacy of in-vivo RIPK3 inhibition was confirmed by immunoblot in Additional file 4: Figure S2A. Unlike the administration of non-specific (NS) siRNA in the Notch1^M−KO^ mice, the knockdown of RIPK3 significantly (*p* < 0.05) decreased IR-induced liver damage (Fig. 4A) and the sALT and sAST levels (Fig. 4B) in the Notch1^M−KO^ mice. RIPK3 siRNA treatment in the Notch1^M−KO^ livers reduced CD11b^+^ macrophage (Fig. 4C) and Ly6G neutrophil (Fig. 4D) accumulation. Moreover, RIPK3 knockdown decreased mRNA levels of TNF-α, IL-1β, CCL-2, and CXCL-10 in Notch1 myeloid knockout mice (Fig. 4E). Notably, RIPK3 siRNA treatment alleviated hepatocyte apoptosis/necroptosis/necrosis evidenced by decreased TUNEL^+^ cells in IR-stressed livers compared to the NS siRNA-treated livers (Fig. 4F). These results demonstrate RIPK3 plays a critical role in the Notch1-mediated immune regulation and hepatocyte apoptosis/ necroptosis/ necrosis in IRI livers.

### β-catenin interacts with NICD and mediates TAK1 activation in macrophages

**Fig. 5:**
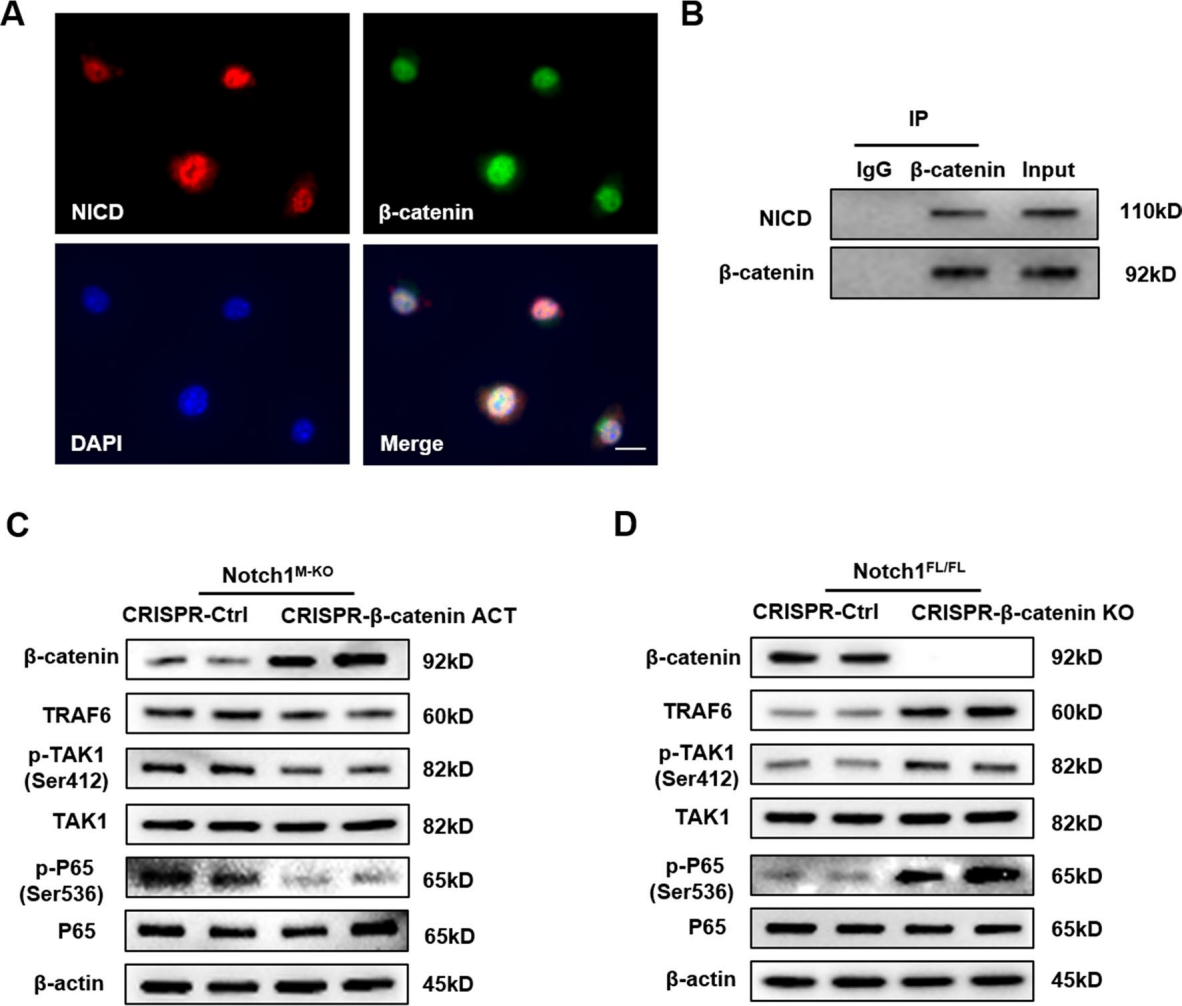
β-catenin interacts with NICD and mediates TAK1 activation in macrophages. Bone marrow-derived macrophages (BMMs, 1 × 10^6^) were cultured with or without LPS (100 ng/ml) for 6 h. **A** Immunofluorescence staining for macrophage β-catenin (green) and NICD (red) co-localization in LPS-stimulated macrophages. DAPI was used to visualize nuclei (blue). Scale bars, 30 μm. **B** Immunoprecipitation analysis of β-catenin and NICD in LPS-stimulated macrophages. **C** Western blot analysis of β-catenin, TRAF6, p-TAK1 and p-P65 in BMMs transfected with CRISPR/Cas9-mediated β-catenin activation and control. Representative of three experiments. **D** Western blot analysis of β-catenin, TRAF6, p-TAK1 and p-P65 in BMMs transfected with CRISPR/Cas9-mediated β-catenin KO and control. Representative of three experiments

Having demonstrated that β-catenin is a key regulator of Notch1-mediated inflammatory response in IR-stress livers, we then analyzed putative crosstalk between β-catenin and the TAK1 pathway in macrophages. Interestingly, Immunofluorescence staining revealed that β-catenin co-localized with NICD in LPS-stimulated BMMs (Fig. 5A). The co-immunoprecipitation assay revealed β-catenin could directly bound to endogenous NICD in BMMs after LPS stimulation (Fig. 5B). To further confirm that β-catenin is required for the crosstalk between Notch1 and TAK1-mediated innate immune responses in vitro, we introduced CRISPR/Cas9. Strikingly, CRISPR/Cas9-mediated β-catenin activation reduced TRAF6, p-TAK1, and p-P65 expression (Fig. 5C) in macrophages from Notch1^M−KO^ mice. Similarly, CRISPR/Cas9-mediated β-catenin KO increased TRAF6, p-TAK1, and p-P65 expression (Fig. 5D) in macrophages from Notch1^FL/FL^ mice. These results indicate that β-catenin interacts with NICD and controls the crosstalk between Notch1 and TAK1-mediated innate immune responses.

### TRAF6 is essential for the Notch1-mediated inflammatory response and ROS generation in LPS-stimulated macrophages

**Fig. 6:**
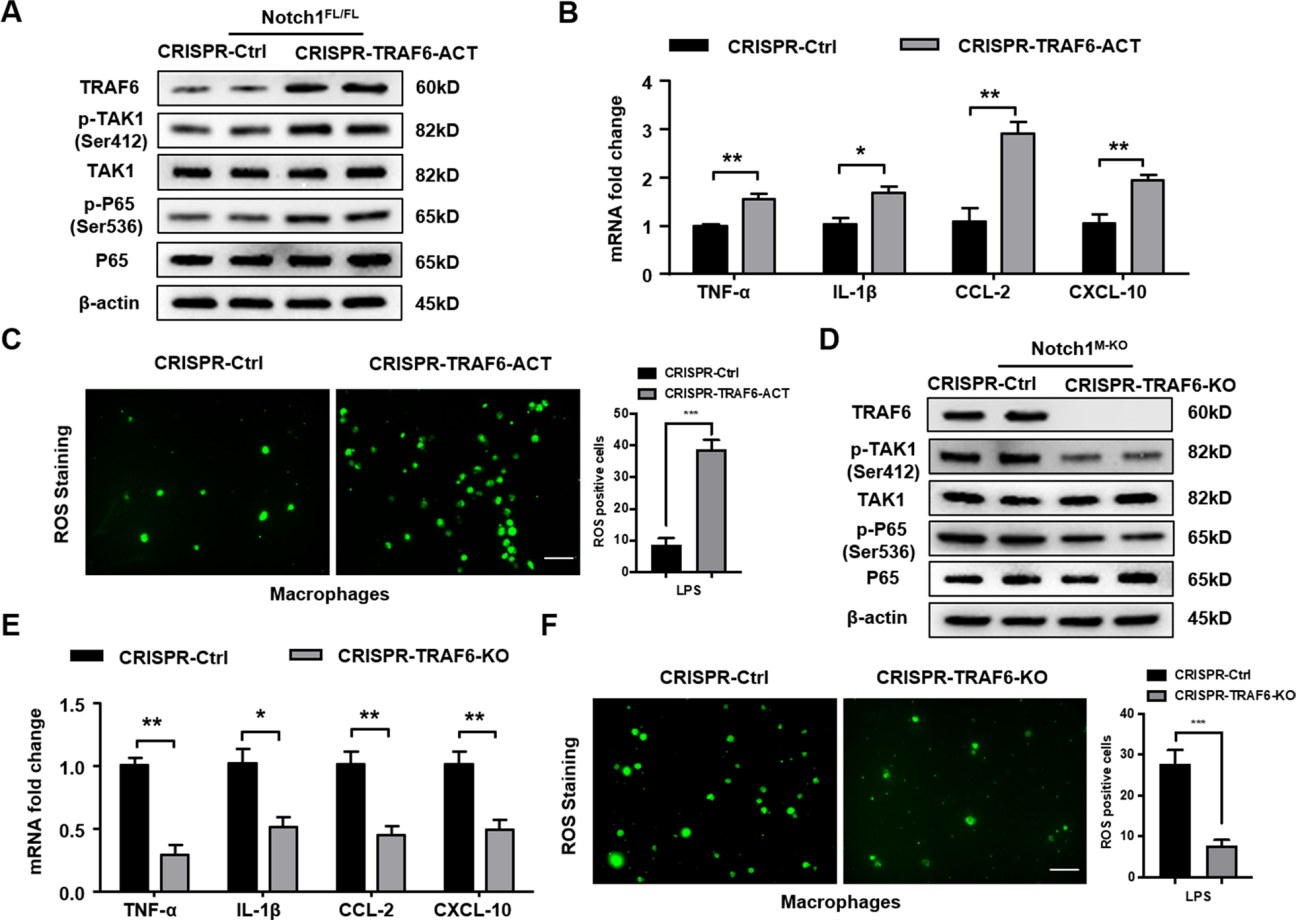
TRAF6 is essential for the Notch1-mediated inflammatory response and ROS generation in LPS-stimulated macrophages. BMMs were isolated from the Notch1^FL/FL^ and Notch1^M−KO^ mice and transfected with CRISPR/Cas9-mediated TRAF6 activation or TRAF6 KO vector. **A** Western blot analysis of TRAF6, p-TAK1 and p-P65 in BMMs transfected with CRISPR/Cas9-mediated TRAF6 activation and control. Representative of three experiments. **B** Quantitative RT-PCR analysis of TNF-α, IL-1β, CCL-2 and CXCL-10 mRNA levels in vitro (n = 3–4 samples/group). **C** Detection of ROS production by Carboxy-H2DFFDA in LPS-stimulated macrophages from the Notch1^FL/FL^ mice. Quantification of ROS-producing Macrophages (green) (n = 4–6 mice/group). Scale bars, 40 μm. **D** Western blot analysis of TRAF6, p-TAK1 and p-P65 in BMMs transfected with CRISPR/Cas9-mediated TRAF6 KO and control. Representative of three experiments. **E** Quantitative RT-PCR analysis of TNF-α, IL-1β, CCL-2 and CXCL-10 mRNA levels in vitro (n = 3–4 samples/group). **F** Detection of ROS production by Carboxy-H2DFFDA in LPS-stimulated macrophages from the Notch1^M−KO^ mice. Quantification of ROS-producing Macrophages (green) (n = 4–6 mice/group). Scale bars, 40 μm. All data represent the mean ± SD. *p < 0.05. **p < 0.01. ***p < 0.001

To further confirm the role of TRAF6 in the regulation of macrophage Notch1-mediated immune responses in IR-stressed liver, BMMs were isolated from the Notch1^FL/FL^ and Notch1^M−KO^ mice and transfected with CRISPR/Cas9-mediated TRAF6 activation or TRAF6 KO vector. Indeed, CRISPR/Cas9-mediated TRAF6 activation increased p-TAK1 and p-P65 expression in Notch1^FL/FL^ macrophages (Fig. 6A), along with augmented mRNA levels coding for TNF-α, IL-1β, CCL-2, and CXCL-10 (Fig. 6B) in Notch1^FL/FL^ macrophages after LPS-stimulation. ROS production presented by Carboxy-H2DFFDA showed enhanced ROS generation in CRISPR-TRAF6 activation macrophages (Fig. 6C). Moreover, CRISPR/Cas9-mediated TRAF6 KO diminished p-TAK1 and p-P65 expression (Fig. 6D). The mRNA levels coding for TNF-α, IL-1β, CCL-2, and CXCL-10 were also reduced (Fig. 6E), accompanied by down-regulated ROS production in Notch1^M−KO^ macrophages after LPS-stimulation (Fig. 6F). These results indicate that TRAF6 is critically involved in activating TAK1-mediated inflammatory responses.

### Macrophage Notch1 deficiency-mediated TRAF6 enhances stress-induced hepatocyte necroptosis via modulating RIPK3 signaling activation

**Fig. 7:**
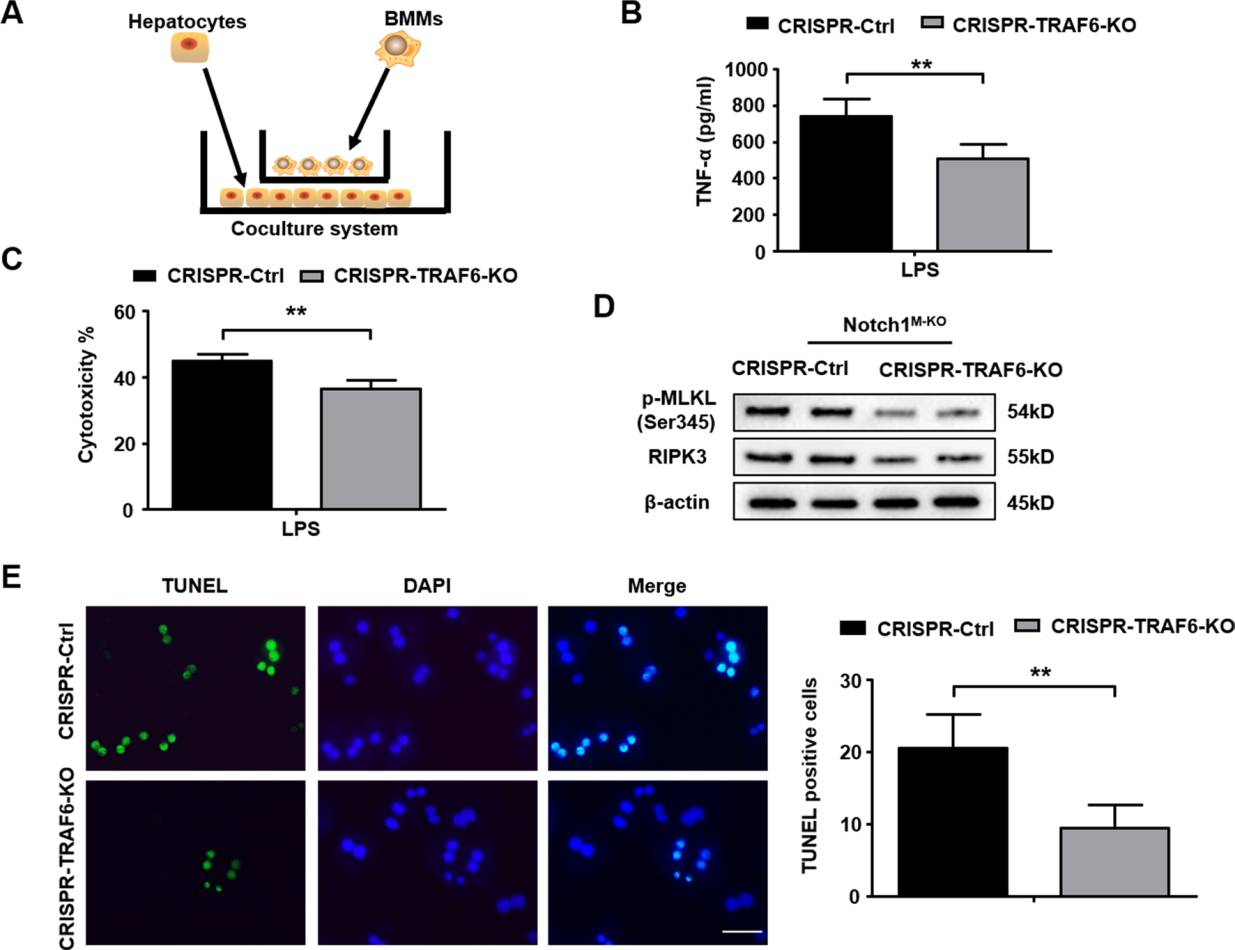
Macrophage Notch1 deficiency-mediated TRAF6 enhances stress-induced hepatocyte necroptosis via modulating RIPK3 signaling activation. BMMs were isolated from Notch1^M−KO^ mice and transfected with the p-CRISPR-TRAF6 KO or control vector followed by LPS stimulation. **A** The schematic figure for macrophage/hepatocyte co-culture system. **B** ELISA analysis of supernatant TNF-α levels in LPS-stimulated BMMs (n = 3–4 samples/group). **C** LDH release from hepatocytes in co-cultures. Quantification of average cytotoxicity (% cell death) in different groups were plotted. (n = 3–4 samples/group). **D** BMMs transfected with the p-CRISPR-TRAF6 KO were stimulated with LPS, and then co-cultured with primary hepatocytes supplemented with or without H_2_O_2_ for 24 h. Western-assisted analysis of p-MLKL and RIPK3 in hepatocytes after co-culture. Representative of three experiments. **E** Immunofluorescence staining of TUNEL^+^ hepatocytes after co-culture (n = 4–6 mice/group). Scale bars, 40 μm. All data represent the mean ± SD. **p < 0.01

We then asked how macrophage TRAF6 may regulate hepatocyte necroptosis under inflammatory conditions. Using a co-culture system with LPS-stimulated CRISPR-TRAF6 KO BMMs from the Notch1^M−KO^ mice and primary hepatocytes isolated from WT mice supplemented with H_2_O_2_ (Fig. 7A), we found decreased TNF-α release from LPS-stimulated Notch1^M−KO^ BMMs after transfection with a CRISPR-TRAF6 KO vector compared to the control groups (Fig. 7B). Moreover, our results showed that the LDH released from stressed hepatocytes was markedly inhibited in the macrophage TRAF6 KO group (Fig. 7C). Unlike the control groups, macrophage TRAF6 KO decreased hepatocyte p-MLKL and RIPK3 expression (Fig. 7D). Strikingly, Immunofluorescence staining revealed reduced TUNEL^+^ hepatocytes after co-culture with the CRISPR-TRAF6 KO BMMs but not the control cells (Fig. 7E). These results indicate that macrophage TRAF6 deficiency diminishes hepatocyte necroptosis by inhibiting RIPK3-pMLKL signaling in response to inflammatory responses.

## Discussion

In this study, we revealed that disruption of macrophage Notch1 activated the TAK1-mediated inflammatory responses and RIPK3-mediated hepatocyte necroptosis through activation of β-catenin in IR stress-induced liver inflammation. In detail, we showed that macrophage Notch1 deficiency inhibited the expression of β-catenin, which led to enhanced TAK1 signaling activation and hepatocyte necroptosis in liver IRI. Our findings demonstrate the macrophage Notch1-β-catenin axis is a crucial regulatory mechanism in IR-triggered liver inflammation and provide novel therapeutic potential in organ IRI and transplant recipients.

The Notch pathway is considered as the most commonly activated pathway in the process of liver inflammatory diseases [21, 22]. As Notch signaling participates in a variety of cellular activities, the modulation of the Notch cascade has been proved to cause lots of pathological changes. For example, the stimulation of the Notch pathway by TLR4 activation may involve gene expression profiles linking with inflammatory processes [23]. Moreover, the Notch intracellular domain (NICD) inhibited proinflammatory cytokine release when expression of the anti-inflammatory regulator was stimulated in TLR-activated macrophages [24]. In addition, Notch1 and one of its target genes Hes1 might suppress the inflammation through transcriptional regulation. RhoA/ROCK signaling was stimulated in myeloid Notch1 deficient mice and aggravated liver inflammation [25]. These results implied the Notch pathway regulates inflammatory responses through multiple mechanisms. This study investigated the roles and molecular mechanisms of macrophage Notch1 in modulating TAK1-mediated innate immune responses in liver IRI. Our findings reveal a novel mechanism in which Notch1 signaling regulates the TAK1-related innate immune response and RIPK3-mediated necroptosis through β-catenin in IR-stressed livers.

TAK1 involves in many crucial processes, such as cell death, immune regulation, and carcinogenesis [26]. Previous studies have reported that the deficiency of TAK1 significantly improved energy metabolism, inflammation, fibrosis, and tumor formation [27–29]. on the other hand, hyperactivation of TAK1 may cause hepatic inflammation, metabolic disturbances, and fibrosis. Thus, TAK1 homeostasis is vital to liver physiological activity [25]. Our results revealed the crosstalk between TAK1-mediated innate immune responses and the Notch1 signaling pathway in liver IRI, proving that the Notch1 pathway could regulate TAK1-mediated inflammatory responses.

However, it remains unclear how Notch1 may modulate TAK1-mediated inflammatory response in IR-stressed liver. In line with our in vitro findings, we showed that lentivirus β-catenin-modified macrophages alleviated IR-induced liver damage and reduced CD11b^+^ macrophage and Ly6G^+^ neutrophil accumulation, along with the decrease of proinflammatory cytokine. Notably, using the immunofluorescence staining and co-immunoprecipitation assay*,* we found that β-catenin co-localized with NICD*.* Indeed, CRISPR/Cas9-mediated β-catenin activation reduced TRAF6, p-TAK1, and p-P65 expression in macrophages from Notch1^M−KO^ mice, and CRISPR/Cas9-mediated β-catenin KO increased TRAF6, p-TAK1, and p-P65 expression in macrophages from Notch1^FL/FL^ mice. Our findings revealed the distinct ability of Notch1 in controlling TAK1 function through β-catenin in IR stress-induced liver injury.

Interestingly, macrophage Notch1-mediated TRAF6 could also be involved in regulating IR-induced necroptosis pathways. IR-stress related ROS may induce RIPK3 stimulation. RIPK3 is an essential regulator in necroptosis. Through the RIP-homotypic interaction motif (RHIM), RIPK3 interacts with its upstream kinase RIPK1 to generate a necrosome, which functions as a recruitment factor of the Mixed lineage kinase domain like pseudo kinase (MLKL) [26]. Activated MLKL also causes membrane rupture. As a result, danger-associated molecular patterns (DAMPs) are released to facilitate inflammatory responses [27]. Indeed, as a critical switch for necrosis and inflammation, RIPK3 activation, which is regulated by caspase-mediated cleavage, promoted inflammation related cell death and injury in different inflammatory disease models [30]. RIPK3 deficiency alleviated liver fibrosis in a caspase-8-dependent manner by regulating JNK signaling pathway [31]. In line with these results, our precious study displayed that disruption of RIPK3 reduced caspase-1 activity by inhibiting NEK7/NLRP3 function in hepatic ischemia–reperfusion injury [32]. The present study showed that CRISPR/Cas9-mediated TRAF6 KO inhibited TNF-α release in Notch1^M−KO^ macrophages under LPS stimulation. Using in vitro macrophage/hepatocyte co-culture system, we showed that macrophage TRAF6 deficiency decreased hepatocyte p-MLKL and RIPK3 expression, and reduced LDH release from H_2_O_2_-treated hepatocytes. In addition, immunofluorescence staining result further identified that macrophage TRAF6 deletion alleviated the hepatocyte necroptosis in H_2_O_2_-induced injury. Our findings demonstrate that macrophage Notch1 deficiency-mediated TRAF6 activation promotes hepatocyte necroptosis through modulation of RIPK3-p-MLKL initiated caspase cascade activation.

## Conclusions

To sum up, we identify a previously unrecognized role of the macrophage Notch1-β-catenin axis in regulating TAK1-mediated innate immune response and RIPK3-mediated necroptosis signaling pathway in IR stress-induced liver injury. We identify that the macrophage Notch1-mediated β-catenin axis controls TAK1-dependent inflammation. By disclosing the molecular regulatory mechanism of macrophage β-catenin-mediated Notch1-TAK1 pathway in IR- stressed liver, our findings provide novel potential therapeutic targets in IRI and liver inflammation.

## Supplementary Information

**Additional file 1.** Supplementary Materials.

**Additional file 2.** Supplementary Table 1.

**Additional file 3.** Supplementary Figure1.

**Additional file 4.** Supplementary Figure2.

## Data Availability

The datasets generated for this study are available on request to the corresponding author.

## Abbreviations

IRI: Ischemia/reperfusion injury
sALT: Serum alanine aminotransferase
ROS: Reactive oxygen species
LPS: Lipopolysaccharide
BMMs: Bone marrow-derived macrophages
CMFDA: 5-Chloromethylfluorescein diacetate
CRISPR: Clustered regularly interspaced short palindromic repeats
Cas9: CRISPR associated protein 9
NICD: Notch1 intracellular domain
PCR: Polymerase chain reaction
TUNEL: Terminal deoxynucleotidyl transferase dUTP nick end labelling
TAK1: Transforming growth factor beta-activated kinase 1
WT: Wild type
RIPK3: Receptor-interacting serine/threonine-protein kinase 3
MLKL: Mixed-lineage kinase domain like protein
