# Supplementary material for: Macrophage Notch1 inhibits TAK1 function and RIPK3-mediated hepatocyte necroptosis through activation of β-catenin signaling in liver ischemia and reperfusion injury
Source: Cell Commun Signal. 2022 Sep 16;20:144. doi: 10.1186/s12964-022-00901-8 (PMC9479434; doi:10.1186/s12964-022-00901-8)
Supplement: Supplementary file 2 — Additional file 1. Supplementary Materials. [file 12964_2022_901_MOESM2_ESM.docx]

**Supplementary Materials**

**Macrophage Notch1 Inhibits TAK1 Function and RIPK3-Mediated Hepatocyte Necroptosis Through Activation of β-Catenin Signaling in Liver Ischemia and Reperfusion Injury**

**Dongwei Xu^1#^, Xiaoye Qu^1#^, Yizhu Tian^2#^,** **Zhao Jie^1#^**, Zhifeng Xi^1^, Feng Xue^1^, Xueyun Ma^3^*, Jianjun Zhu^1^*, Qiang Xia^1^*

1.Department of Liver Surgery, Renji Hospital, Shanghai Jiaotong University School of Medicine, Shanghai 200127, China

2.Department of General Surgery, Pukou Branch of Jiangsu Province hospital, Nanjing Medical University, Nanjing 211166, China

3.Shanghai key laboratory of Regulatory Biology, Institude of Biomedical Sciences and School of life Sciences, East China Normal University, Shanghai 200241, China.

^#^ These authors contributed equally to this work.

**Experimental Procedures**

**Animals**

The floxed Notch1 (Notch1^FL/FL^) mice (The Jackson Laboratory, Bar Harbor, ME) and the mice expressing Cre recombinase under the control of the Lysozyme 2 (Lyz2) promoter (LysM-Cre; The Jackson Laboratory) were used to generate myeloid-specific Notch1 knockout (Notch1^M-KO^) mice. Two steps were used to create Notch1^M-KO^ mice. First, a homozygous loxP-flanked Notch1 mouse was mated with a homozygous Lyz2-Cre mouse to generate the F1 mice that were heterozygous for a loxP-flanked Notch1 allele and heterozygous for the Lyz2-Cre. Next, these F1 mice were backcrossed to the homozygous loxP-flanked Notch1 mice, resulting in the generation of Notch1^M-KO^ (25% of the offspring), which were homozygous for the loxP-flanked Notch1 allele and heterozygous for the Lyz2-Cre allele. Mouse genotyping was performed by using a standard protocol with primers described in the JAX Genotyping protocols database. Male C57BL/6 wild-type (WT) mice were obtained from The Jackson Laboratory. Animals at 6-8 weeks of age were used in all experiments. All animal studies were approved by the Institutional Animal Care and Use Committees of Renji Hospital and Shanghai Jiaotong University.

**Mouse liver IRI model**

We used an established model of mice 70% warm hepatic ischemia (90min) followed by reperfusion (6h). Briefly, mice were injected with heparin (100U/kg), and an atraumatic clip was applied to interrupt the arterial/portal venous blood supply to the cephalad liver lobes. The clip was removed after 90min of ischemia. The mice were sacrificed at 6h of reperfusion(1). Some mice have been injected with the bone marrow-derived macrophages (BMMs, 5×10^6^ cells in PBS/mouse) transfected with lentivirus-expressing β-catenin (Lv-β-catenin) or control lentivirus (GFP) via the tail vein 24h before ischemia. The Lv-β-catenin was kindly provided by Dr. Bibo Ke (University of California, Los Angeles)(2). Some animals were injected with RIPK3 siRNAs (sc-61483) or non-specific (NS) control siRNA (2mg/kg) (Santa Cruz Biotechnology, Santa Cruz, CA) mixed with mannose-conjugated polymers (Polyplus transfection™, Illkirch, France) according to the manufacturer’s instructions 4h before ischemia.

**Hepatocellular function assay**

The serum alanine aminotransferase (sALT) and aspartate aminotransferase (AST) were measured by ALT and AST kit (ThermoFisher, Waltham, MA) according to the manufacturer’s instructions.

**Histology, immunohistochemistry, and immunofluorescence staining**

Liver sections (5-μm) were stained with hematoxylin and eosin (H&E). The severity of IRI was graded using Suzuki’s criteria(3). Liver macrophages were indicated by primary CD11b+ rat monoclonal antibodies (mAb) (Abcam, Cambridge, MA) and secondary Cy5-conjugated AffiniPure donkey anti-rat IgG (Jackson Immunoresearch, West Grove, PA) for immunofluorescence staining. The neutrophils were measured using primary Ly6G rat mAb (Invitrogen, San Diego, CA) by immunohistochemistry (IHC) staining. The RIPK3 in the mice liver was detected by IHC using RIPK3 mAb (Abcam, Cambridge, MA). The primary mouse NICD (Santa Cruz Biotechnology) mAb and rabbit β-catenin (Cell Signaling Technology) mAb, the secondary AlexFluor488-conjugated AffiniPure donkey anti-rabbit IgG Ab, Cy5-conjugated AffiniPure donkey anti-mouse IgG Ab (Jackson Immunoresearch) were used for the double immunofluorescence staining according to the manufacturer’s instructions. Images for immunofluorescence staining were captured using a fluorescence microscope (Keyence BZ-X810, Osaka, Japan) and analyzed using Image-pro Plus software. Positive cells were counted blindly in 10 HPF/section (x200).

**Quantitative RT-PCR analysis**

Quantitative real-time PCR was performed as described(4). Total RNA was purified from liver tissue or cell cultures using RNeasy Mini Kit (Qiagen, Chatsworth, CA) according to the manufacturer’s instructions. Reverse transcription to cDNA was performed by using SuperScript III First-Strand Synthesis System (ThermoFisher Scientific). Quantitative real-time PCR was carried out using the QuantStudio 3 (Applied Biosystems by ThermoFisher Scientific). In a final reaction volume of 25μl, the following were added: 1x SuperMix (Platinum SYBR Green qPCR Kit; Invitrogen) cDNA and 10μM of each primer. Amplification conditions were: 50°C (2min), 95°C (5min), followed by 40 cycles of 95°C (15sec) and 60°C (30sec). Primer sequences used to amplify TNF-α, IL-1β, CCL-2, and CXCL-10 were shown in Supplementary Table 1. Target gene expressions were calculated by their ratios to the housekeeping gene β-actin.

**Western blot analysis**

Protein was extracted from liver tissue or cell cultures as described(5). Protein was extracted from liver tissue or cell cultures with ice-cold protein lysis buffer (50mM Tris, 150mM NaCl, 0.1% sodium dodecyl sulfate, 1% sodium deoxycholate, 1% Triton-100). The buffer contains 1% proteinase and phosphatase inhibitor cocktails (Sigma-Aldrich, St. Louis, MO). Proteins (30 µg/sample) in SDS-loading buffer (50mM Tris, pH 7.6, 10% glycerol, 1% SDS) were subjected to 4-20% SDS-polyacrylamide gel electrophoresis (PAGE) and transferred to nitrocellulose membrane (Bio-Rad). The membrane was blocked with 5% dry milk and 0.1% Tween 20 (USB, Cleveland, OH). The nuclear and cytosolic fractions were prepared with NE-PER Nuclear and Cytoplasmic Extraction Reagents (ThermoFisher Scientific). The NICD, Bax, Bcl-2, c-caspase-3, TRAF6, p-TAK1, TAK1, p-P65, P65, RIPK3, p-MLKL, Lamin B2, β-actin (Cell Signaling Technology) antibodies were used. The membranes were incubated with Abs, and then added Western ECL substrate mixture (Bio-Rad) for imaging with the iBright FL1000 (ThermoFisher Scientific). Relative quantities of protein were determined by comparing the β-actin expression using iBright image analysis software (ThermoFisher Scientific).

**Isolation of primary hepatocytes, Kupffer cells, and bone marrow-derived macrophages**

Primary hepatocytes, Kupffer cells, and BMMs from the Notch1^FL/FL^, Notch1^M-KO^, or wild-type (WT) mice were isolated as described(5, 6). Briefly, livers were perfused in situ with warmed (37℃) HBSS solution, followed by a collagenase buffer (collagenase type IV, SigmaAldrich). Perfused livers were dissected and teased through 70-μm nylon mesh cell strainers (BD Biosciences, San Jose, CA). Nonparenchymal cells (NPCs) were separated from hepatocytes by centrifuging at 50 × g for 2min three times. NPCs were suspended in HBSS and layered onto a 50%/25% two-step Percoll gradient (Sigma) in a 50-ml conical centrifuge tube and centrifuged at 1800 × g at 4°C for 15min. Kupffer cells in the middle layer were collected and attached to cell culture plates in DMEM with 10% FBS, 10mM HEPES, 2mM GlutaMax, 100 U/ml penicillin, and 100 μg/ml streptomycin for 15min at 37°C. Nonadherent cells were removed by replacing the culture medium. Murine bone-derived macrophages (BMMs) were generated as described(7, 8). In brief, bone marrow cells were removed from the femurs and tibias of the Notch1^FL/FL^, Notch1^M-KO^, or WT mice and cultured in DMEM supplemented with 10% FCS and 15% L929-conditioned medium for seven days.

**Co-culture of macrophages and primary hepatocytes.**

The co-culture system was conducted as described(5). In brief, primary hepatocytes were cultured in 6-well plates at a concentration of 4x10^5^ cells per well. After 24h, the 0.4μm-pore size transwell inserts (Corning) containing 1x10^6^ BMMs were placed into the 6-well plate with the initially seeded hepatocytes. Co-cultures were incubated for 12h with or without adding H_2_O_2_ (200 µM) in the lower chamber.

**ELISA assay**

The cell culture supernatants were harvested for cytokine analysis. The ELISA kit (ThermoFisher Scientific) was used to measure TNF-α levels according to the manufacturer’s instructions.

**LDH activity assay**

BMMs (1x10^6^) were cultured with primary hepatocytes (4x10^5^ /well) for 12h with or without adding H_2_O_2_ (200 µM) in the lower chamber. The activity of lactate dehydrogenase (LDH) in the cell culture medium from the lower chamber was measured with a commercial LDH activity assay kit (Stanbio Laboratory, Boerne, TX) according to manufacturer’s instructions.

**TUNEL assay**

The DNA fragmentation characteristic of apoptotic cells in formalin-fixed paraffin-embedded liver sections was detected with an in situ cell death detection kit (Roche) according to the manufacturer’s instructions. Apoptosis of primary hepatocytes was measured by Cell Meter TUNEL Apoptosis Assay Kit (AAT Bioquest, Sunnyvale, CA). Briefly, treated hepatocytes were fixed in 4% paraformaldehyde for 30 min. After three washes with TBST, hepatocytes were incubated with Tunnelyte TM Green for 60 min at 37˚C. Additional Hoechst staining was conducted for the nucleus identification. Results were scored semi-quantitatively by averaging the number of apoptotic cells/microscopic field at 200x magnification. Ten fields were evaluated per sample.

**Reactive oxygen species assay**

ROS production in Kupffer cells was measured using the 5-(and-6)-carboxy-2',7'-difluoro dihydro fluorescein diacetate (Carboxy-H2DFFDA, ThermoFisher Scientific) according to the manufacturer’s instructions. In brief, Kupffer cells were cultured on collagen-coated coverslips after LPS stimulation. After washing with PBS, cells were then incubated with 10μM of Carboxy-H2DFFDA. The Carboxy-H2DFFDA was converted to a green-fluorescent form when hydrolyzed by intracellular esterase and oxidized in the cells. ROS produced by Kupffer cells were analyzed by fluorescence microscopy. Positive green fluorescent-labeled cells were counted blindly in 10 HPF/section (x200).

**In vitro transfection**

Cells (1x10^6^/well) were cultured for seven days and then and then transfected with CRISPR/Cas9-β-catenin KO (sc-419477-KO), CRISPR-β-catenin activation (sc-419477-ACT), CRISPR/Cas9-TRAF6 KO (sc-423497), CRISPR-TRAF6 activation (sc-423497-ACT) or control vector (Santa Cruz Biotechnology) by using Lipofectamine TM 3000 according to the manufacturer’s instructions (ThermoFisher Scientific). After 24-48h, cells were supplemented with 100 ng/ml of lipopolysaccharide (LPS) for an additional 6h.

**Immunoprecipitation analysis**

Immunoprecipitation and immunoblotting were performed as described previously(1). The Pierce™ classic magnetic IP/Co-IP Kit (Thermo Fisher, 88804) was applied for immunoprecipitation and immunoblotting. Briefly, BMMs after LPS stimulation were lysed in NP-40 lysis buffer (50mM Tris pH7.4, 10 mM EDTA, 150 mM NaCl, 1% NP-40, ThermoFisher Scientific) containing protease inhibitors. The lysates were incubated with β-catenin (Cell Signaling Technology) mAb, or control IgG and protein A/G beads at 4 ^o^C overnight. After immunoprecipitation, the immunocomplexes were washed with lysis buffer three times. The primary mouse NICD (Santa Cruz Biotechnology) mAb was used for the standard immunoblot procedures according to manufacturer’s instructions.

**Supplemental Figures**

**Figure S1**


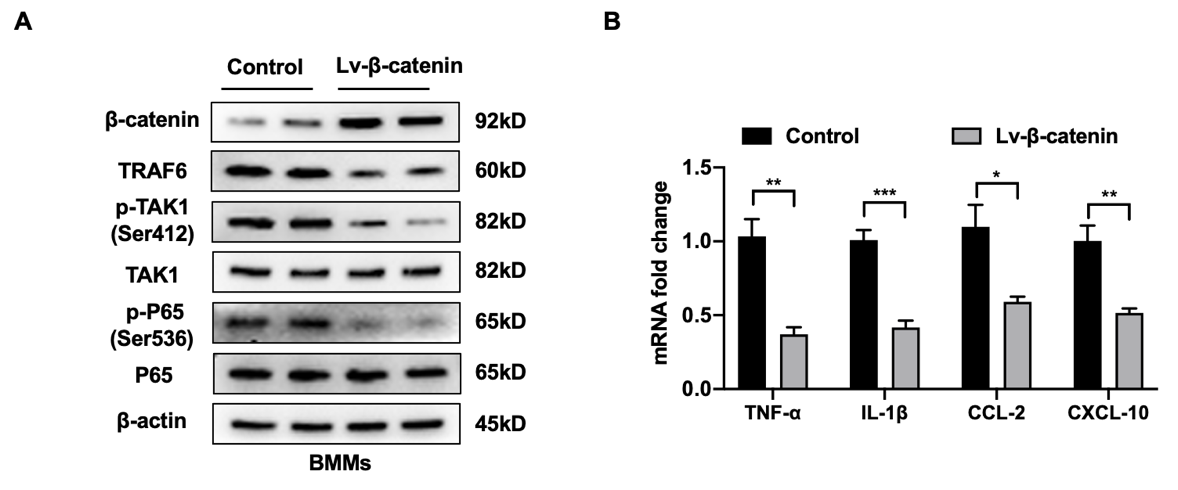


**Supplemental figure 1.** **The effect of β-catenin overexpression in BMMs isolated from** **Notch1^M-KO^ mice.**

(A) Western blot analysis of β-catenin, TRAF6, p-TAK1, and p-P65. Representative of three experiments. (B) Quantitative RT-PCR analysis of TNF-α, IL-1β, CCL-2 and CXCL-10 mRNA levels in BMMs (n=3-4 samples/group). All data represent the mean*±* SD. *p<0.05. **p<0.01. ***p<0.001.

**Figure S2**

**
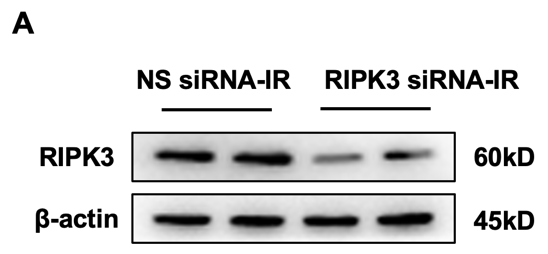
**

**Supplemental figure 2. The** **efficacy of** **in-vivo RIPK3 inhibition.**

(A) Western blot analysis of RIPK3 in the Notch1^M-KO^ mice which were injected with RIPK3 siRNA (2 mg/kg) or non-specific (NS) control siRNA mixed with mannose-conjugated polymers. Representative of three experiments.

**References**

1. Sheng M, Lin Y, Xu D, Tian Y, Zhan Y, Li C, Farmer DG, et al. CD47-Mediated Hedgehog/SMO/GLI1 Signaling Promotes Mesenchymal Stem Cell Immunomodulation in Mouse Liver Inflammation. Hepatology 2021;74:1560-1577.

2. Yue S, Zhu J, Zhang M, Li C, Zhou X, Zhou M, Ke M, et al. The myeloid heat shock transcription factor 1/β-catenin axis regulates NLR family, pyrin domain-containing 3 inflammasome activation in mouse liver ischemia/reperfusion injury. Hepatology (Baltimore, Md.) 2016;64:1683-1698.

3. Suzuki S, Toledo-Pereyra LH, Rodriguez FJ, Cejalvo D. Neutrophil infiltration as an important factor in liver ischemia and reperfusion injury. Modulating effects of FK506 and cyclosporine. Transplantation 1993;55:1265-1272.

4. Li C, Sheng M, Lin Y, Xu D, Tian Y, Zhan Y, Jiang L, et al. Functional crosstalk between myeloid Foxo1-β-catenin axis and Hedgehog/Gli1 signaling in oxidative stress response. Cell Death Differ 2021;28:1705-1719.

5. Jin Y, Li C, Xu D, Zhu J, Wei S, Zhong A, Sheng M, et al. Jagged1-mediated myeloid Notch1 signaling activates HSF1/Snail and controls NLRP3 inflammasome activation in liver inflammatory injury. Cell Mol Immunol 2020;17:1245-1256.

6. Lu L, Yue S, Jiang L, Li C, Zhu Q, Ke M, Lu H, et al. Myeloid Notch1 deficiency activates the RhoA/ROCK pathway and aggravates hepatocellular damage in mouse ischemic livers. Hepatology 2018;67:1041-1055.

7. Ke B, Shen XD, Gao F, Ji H, Qiao B, Zhai Y, Farmer DG, et al. Adoptive transfer of ex vivo HO-1 modified bone marrow-derived macrophages prevents liver ischemia and reperfusion injury. Mol Ther 2010;18:1019-1025.

8. Stanley ER, Heard PM. Factors regulating macrophage production and growth. Purification and some properties of the colony stimulating factor from medium conditioned by mouse L cells. J Biol Chem 1977;252:4305-4312.
