# Supplementary material for: Macrophage Notch1 inhibits TAK1 function and RIPK3-mediated hepatocyte necroptosis through activation of β-catenin signaling in liver ischemia and reperfusion injury
Source: Cell Commun Signal. 2022 Sep 16;20:144. doi: 10.1186/s12964-022-00901-8 (PMC9479434; doi:10.1186/s12964-022-00901-8)
Supplement: Supplementary file 3 — Additional file 2. Supplementary Table 1. [file 12964_2022_901_MOESM3_ESM.docx]

| **Supplementary Table 1**: Primer sequences for the amplification | | |
| --- | --- | --- |
| **Target genes** | **Forward primers** | **Reverse primers** |
| **β-actin** | 5’- GTGACGTTGACATCCGTAAAGA-3’ | 5’- GCCGGACTCATCGTACTCC-3’ |
| **TNF-α** | 5’- ACGGCATGGATCTCAAAGAC-3’ | 5’- AGATAGCAAATCGGCTGACG-3’ |
| **IL-1β** | 5’- TGTAATGAAAGACGGCACACC-3’ | 5’- TCTTCTTTGGGTATTGCTTGG-3’ |
| **CCL-2** | 5'-GAAGGAATGGGTCCAGACAT-3' | 5'-ACGGGTCAACTTCACATTCA-3' |
| **CXCL-10** | 5’-GCTGCCGTCATTTTCTGC-3’ | 5’-TCTCACTGGCCCGTCATC-3’ |
