# Supplementary figures and images for: Macrophage Notch1 inhibits TAK1 function and RIPK3-mediated hepatocyte necroptosis through activation of β-catenin signaling in liver ischemia and reperfusion injury
Source: Cell Commun Signal. 2022 Sep 16;20:144. doi: 10.1186/s12964-022-00901-8 (PMC9479434; doi:10.1186/s12964-022-00901-8)

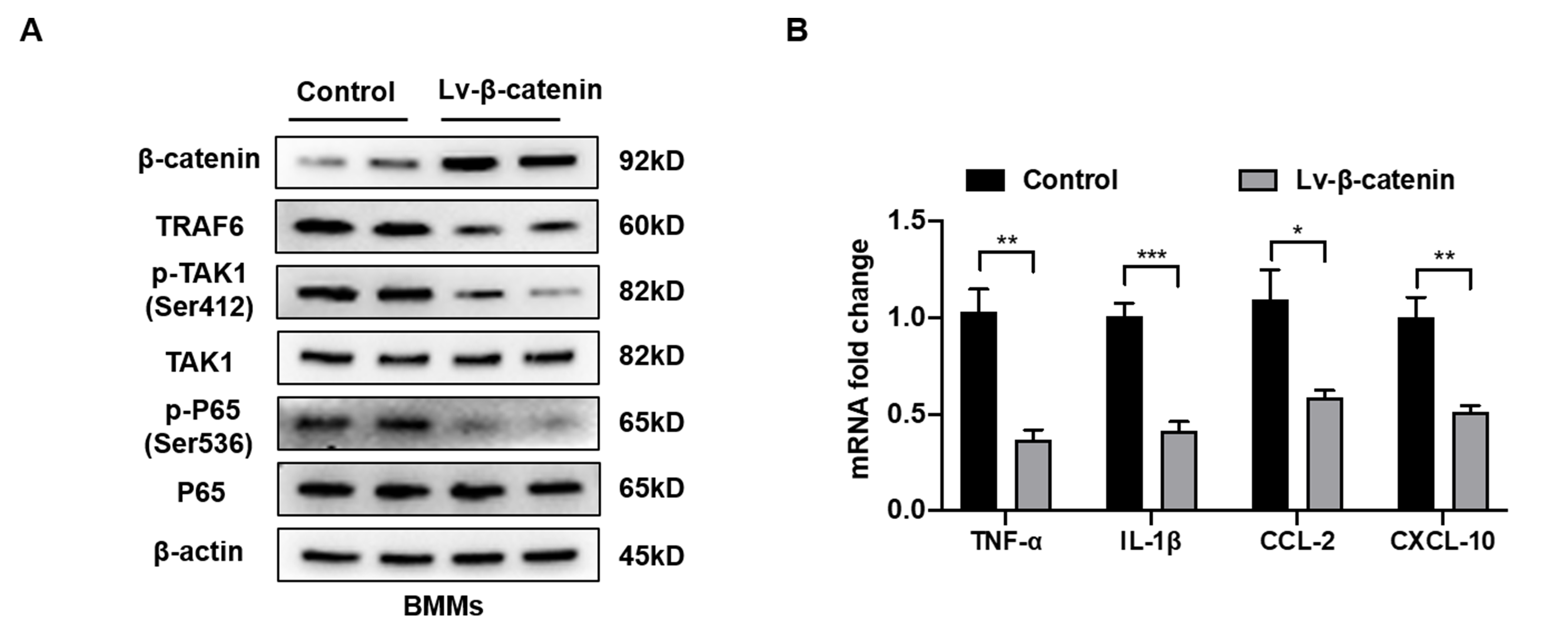

Supplement: Supplementary file 4 — Additional file 3. Supplementary Figure1. [file 12964_2022_901_MOESM4_ESM.tif]

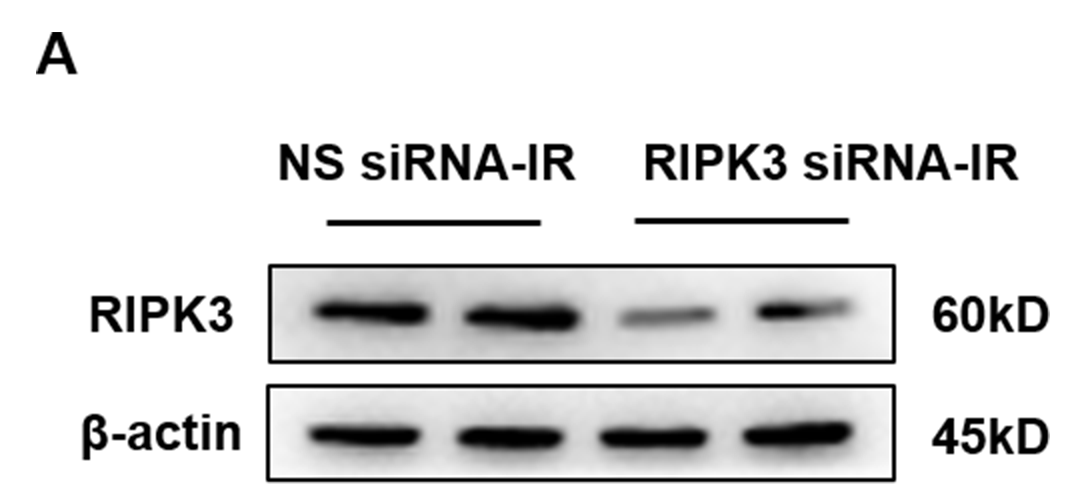

Supplement: Supplementary file 5 — Additional file 4. Supplementary Figure2. [file 12964_2022_901_MOESM5_ESM.tif]
